# Increased expression of IL1-RL1 is associated with type 2 and type 1 immune pathways in asthma

**DOI:** 10.1186/s12865-022-00499-z

**Published:** 2022-05-16

**Authors:** Changyi Xu, Lijuan Du, Fengjia Chen, Kun Tang, Lu Tang, Jia Shi, Lisha Xiao, Zhimin Zeng, Yuxia Liang, Yubiao Guo

**Affiliations:** 1 Department of Pulmonary and Critical Care Medicine, The First Affiliated Hospital of Sun Yat-Sen University, No. 58 Zhongshan 2nd Road, Guangzhou, 510080 Guangdong China; 2 Institute of Respiratory Diseases of Sun Yat-Sen University, No. 58 Zhongshan 2nd Road, Guangzhou, 510080 Guangdong China

**Keywords:** Asthma, IL1-RL1, Induced sputum, Th2 immune, Th1 immune

## Abstract

**Background:**

Asthma is a common chronic airway disease in the world. The purpose of this study was to explore the expression of IL1-RL1 in sputum and its correlation with Th1 and Th2 cytokines in asthma.

**Methods:**

We recruited 132 subjects, detected IL1-RL1 protein level in sputum supernatant by ELISA, and analyzed the correlation between the expression level of IL1-RL1 and fraction of exhaled nitric oxide (FeNO), IgE, peripheral blood eosinophil count (EOS#), and Th2 cytokines (IL-4, IL-5, IL-10, IL-13, IL-33 and TSLP) and Th1 cytokines (IFN-γ, IL-2, IL-8). The diagnostic value of IL1-RL1 was evaluated by ROC curve. The expression of IL1-RL1 was further confirmed by BEAS-2B cell in vitro.

**Results:**

Compared with the healthy control group, the expression of IL1-RL1 in sputum supernatant, sputum cells and serum of patients with asthma increased. The AUC of ROC curve of IL1-RL1 in sputum supernatant and serum were 0.6840 (*p* = 0.0034), and 0.7009 (*p* = 0.0233), respectively. IL1-RL1 was positively correlated with FeNO, IgE, EOS#, Th2 cytokines (IL-4, IL-5, IL-10, IL-13, IL-33 and TSLP) and Th1 cytokines (IFN-γ, IL-2, IL-8) in induced sputum supernatant. Four weeks after inhaled glucocorticoids (ICS) treatment, the expression of IL1-RL1 in sputum supernatant and serum was increased. In vitro, the expression of IL1-RL1 in BEAS-2B was increased after stimulated by IL-4 or IL-13 for 24 h.

**Conclusion:**

The expression of IL1-RL1 in sputum supernatant, sputum cells and serum of patients with asthma was increased, and was positively correlated with some inflammatory markers in patients with asthma. IL1-RL1 may be used as a potential biomarker for the diagnosis and treatment of asthma.

**Supplementary Information:**

The online version contains supplementary material available at 10.1186/s12865-022-00499-z.

## Introduction

Asthma is a chronic airway inflammatory disease involving many inflammatory cells and inflammatory factors, accompanied by airway hyperreaction and airway remodeling. The mechanism of airway inflammation in asthma is complicated. Antonella Muraro divided asthma into two ‘endotypes’: type 2 asthma and non-type 2 asthma [1]. Between them, type 2 asthma is closely related to peripheral blood eosinophil count (EOS#), peripheral blood total IgE, airway hyperreaction, high secretion of mucus and airway remodeling. Its cellular mechanism involves Th2 cytokines, which play an important role in the occurrence and development of asthma.

IL1-RL1 is a specific receptor of IL33, it’s expressed on the surface of many kinds of immune cells [2]. Its gene is encoded in Th2 cells and regulatory T cell receptors, and is one of the selective markers of Th2 cells [3]. IL1-RL1 gene is located on human chromosome 2q12.1 and is about 40 kb in length [4]. ST2 isoforms are transcription products of the IL1-RL1 gene, of which two are reported to be the most important: transmembrane bound isoform (ST2L or ST2 receptor) and truncated soluble isoform (sST2). IL-33 acts as an alarm, alerting the immune system through its receptors, and sST2 is the receptor that binds to IL-33 to work [5]. A genome-wide association study by Moffatt's team involving 10,365 patients with asthma and 16,110 normal controls found that there was a significant genomic correlation between the single-nucleotide polymorphisms of IL-33 and IL1-RL1 and asthma [6]. Similar conclusions have been confirmed in other studies [7, 8]. Therefore, IL1-RL1 may play an important role in asthma. In recent years, more and more studies have found that IL1-RL1 was elevated in patients with asthma, and many experimental results showed that IL1-RL1 was related to the signal of type 2 inflammation. IL-33 drives type 2 response through signal transduction induced by its receptor IL1-RL1 in immune cells, which leads to the production of type 2 cytokines and chemokines [4]. However, at present, the research on the expression of IL1-RL1 in induced sputum of patients with asthma is still very limited.

Here, we explored the expression of IL1-RL1 in induced sputum supernatant, induced sputum cells and serum of patients with asthma, compared the changes of IL1-RL1 in patients with asthma after 4 weeks of ICS treatment, and analyzed the correlation between the expression of IL1-RL1 and Th1 cytokines and Th2 cytokines in patients with asthma.

## Methods

### Participants and inclusion criteria

A total of 132 subjects (30 healthy controls and 102 asthma patients) were enrolled in this study, all of them were from the First Affiliated Hospital of Sun Yat-sen University. Patients with asthma met the diagnostic criteria of the Global Initiative for Asthma (GINA) [9] guidelines and excluded other respiratory diseases. The detailed inclusion and exclusion criteria was shown in Additional file 1: Table S1. Among them, 30 patients were followed up after four weeks treatment. Written informed consent was obtained from all participants and all human-related studies were approved by the Ethics Committee of the First Affiliated Hospital of Sun Yat-sen University. The basic clinical information of the subjects was shown in Table 1.

**Table 1:** Characteristics of subjects

	Healthy subjects	Asthma patients	*p* value
Number	30	102	
Sex, M:F (%F)	11/19 (63.3)	54/48 (47.1)	0.147
Age, yr	32.33 ± 14.33	44.19 ± 15.87	0.0002
BMI, kg/m^2^	21.06 ± 2.804	23.24 ± 3.246	0.0005
*Lung function*			
FEV1, % predicted	102.3 ± 8.367	78.67 ± 24.47	< 0.0001
Histamine PC20, mg/ml	Na	1.862 ± 2.183	-
FeNO, ppb	15.04 ± 7.191	62.44 ± 53.61	< 0.0001
*Blood*			
Eosinophil rate, n (%)	1.821 ± 0.8910	4.637 ± 3.861	0.0002
Neutrophil rate, n (%)	59.67 ± 10.90	60.17 ± 11.90	0.5894
IgE, IU/mL	92.66 ± 103.9	297.0 ± 435.7	0.002

### Sample collection

Induced sputum and peripheral blood were collected from the subjects. The participants coughed deep sputum induced by hypertonic saline, and 10 ml peripheral blood was extracted from participants. When we collected the induced sputum, we selected sputum suppository to weigh, add 4 times the weight of 0.1%DTT to dissolve it [10]. Then the liquid was filtered through the cell sieve. After centrifugation, the supernatant of sputum was separated for ELISA detection, and 1 ml TRIzol were added to the remaining cells for further RNA extraction. As the same, the serum was separated and detected by ELISA kit.

### Determination of FeNO

According to the recommendations of the American Thoracic Society/European Respiratory Society, we used an electrochemical analyzer (NIOX MINO Analytical Instruments, Aerocrine AB, Solna, Sweden) to determine FeNO [11–13]. During the measurement, the subjects were instructed to inhale NO-free air to reach total vital capacity, and then immediately exhale completely to the device at a constant expiratory flow rate of about 50 ml/s for 10 s.

### ICS treatment

Patients with asthma were treated with ICS (budesonide, 160 µg, twice a day). We instruct them to use inhaled drugs correctly. After one month of ICS treatment, the patients who insisted on medication were followed up, and the induced sputum and peripheral blood were collected again.

### Enzyme linked immunosorbent assay (ELISA)

According to the manufacturer's instructions, the protein levels of IL1-RL1, IL-4, IL-5, IL-10, IL-2, IL-8, IL-13, IFN-γ, MUC5AC, IL-33 and TSLP were detected by commercial ELISA kit (MEIMIAN, Wuhan, China). All the samples have been diluted five times. The IL1-RL1 we measured in this study was the soluble form sST2.

### Cell culture and treatment

BEAS-2B cells (ATCC, USA) were cultured in DMEM-high glucose medium (GIBCO, Grand Island, NY, USA) with 1% penicillin–streptomycin (HyClone, Utah, USA) and 10% fetal bovine serum (FBS, ThermoFisher, Massachusetts, USA). The cells were treated with or without 10 ng/ml and 20 ng/ml IL-4/IL-13 (PeproTech, RockyHill, USA) for 24 h.

### Quantitative reverse transcription polymerase chain reaction (qRT-PCR)

Total RNA from induced sputum cells and cultured cells was extracted by TRIzol (Invitgen, Carlsbad, USA) and reverse transcribed into cDNA by the PrimeScript RT reagent kit (Takara, Tokyo, Japan). Roche LightCycler480II real-time system (Basel, Switzerland) and SYBR PreMix Ex Taq (Takara, Dalian, China) were used for qRT-PCR to quantify the mRNA level of IL1-RL1 (Forward: GAAAACCTAGTTACACCGTGGAT, Reverse: GCAAACACACGATTTCTTTCCTG). The reverse transcription parameters were as follows: 37 °C for 15 min, 85 °C for 5 s, and 4 °C for 1 min. The qRT-PCR parameters were: 95 °C for 30 s, followed by 40 cycles of 95 °C for 5 s, then 60 °C for 30 s. GAPDH was used to normalized (Forward: ACCCAGAAGACTGTGGATGG, Reverse: TTCTAGACGGCAGGTCAGGT). The data was analyzed by the 2-ΔΔCt method and reported as the relative variation (log2 transformed).

### Statistical analysis

All the data in this study was analyzed by GraphPad Prism 8.0 (GraphPad, San Diego, California, USA). We used mean ± SD and paired or unpaired t test for normally distributed data, and we expressed as medians with interquartile ranges and used nonparametric tests (Kruskal–Wallis test) for non-normally distributed data. We used Fisher’s exact test to analyze categorical data and used Spearman’s rank-order correlation for correlation analysis. Receiver operating characteristic (ROC) was generated to determine the diagnostic value. *p* < 0.05 was considered statistical significance.

## Results

### Basic information of subjects

A total of 132 subjects were recruited, including 102 asthma patients and 30 healthy participants. Patients with asthma have lower lung function and higher FeNO and IgE. The basic clinical information of the subjects is shown in the Table 1.

Values were presented as mean ± SD. FeNO, fraction of exhaled nitric oxide; FEV1, forced expiratory volume in the first second; PC20, the dose at which FEV1 falls > 20% of baseline FEV1, respectively.

### Expression of IL1-RL1 in induced sputum supernatant of patients with asthma

**Fig. 1:**
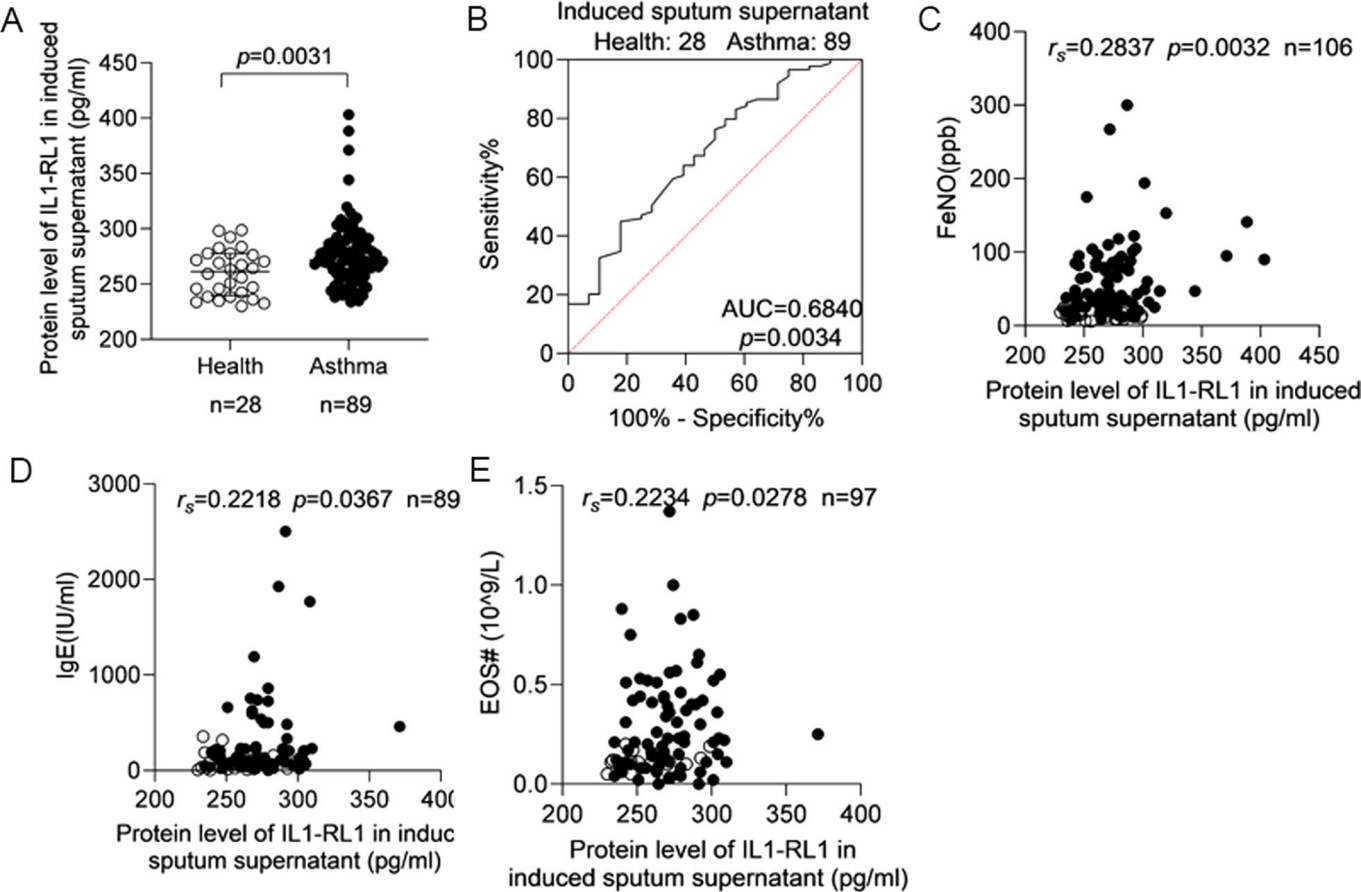
Induced sputum supernatant IL1-RL1 is increased and correlates with FeNO, IgE and EOS# in asthma. **A** The protein level of IL1-RL1 in induced sputum supernatant. **B** The ROC curve of IL1-RL1 in induced sputum supernatant. The relationship between the protein level of IL1-RL1 in induced sputum supernatant and **C** FeNO; **D** IgE; **E** EOS#

The protein level of IL1-RL1 in the supernatant of sputum was detected by ELISA. The results showed that the protein level of IL1-RL1 in the induced sputum supernatant of patients with asthma was higher than healthy controls (*p* = 0.0031) (Fig. 1A). And the AUC in induced sputum supernatant was 0.6840 (*p* = 0.0034) (Fig. 1B). Moreover, the expression of IL1-RL1 in the supernatant of sputum was positively correlated with FeNO (*r*_*s*_ = 0.2837, *p* = 0.0032), IgE (*r*_*s*_ = 0.2218, *p* = 0.0367), EOS# (*r*_*s*_ = 0.2234, *p* = 0.0278) (Fig. 1C–E).

### The relationship between IL1-RL1 and type 2 inflammation

**Fig. 2:**
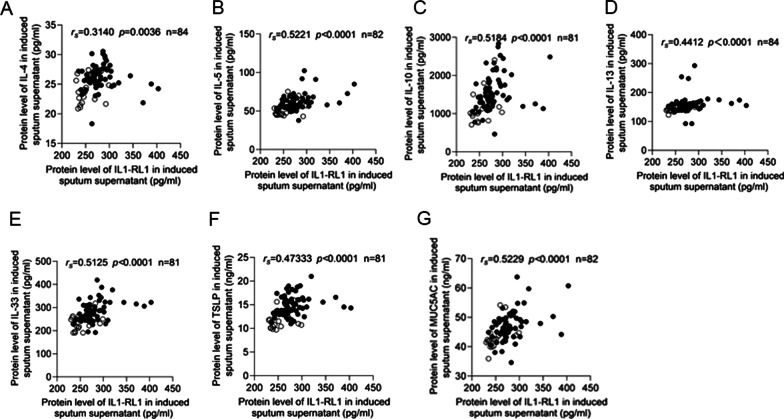
The relationship between IL1-RL1 and type 2 inflammation. The relationship between the protein level of IL1-RL1 in induced sputum supernatant and **A** IL-4; **B** IL-5; **C** IL-10; **D** IL-13; **E** IL-33; **F** TSLP and **G** MUC5AC

**Fig. 3:**
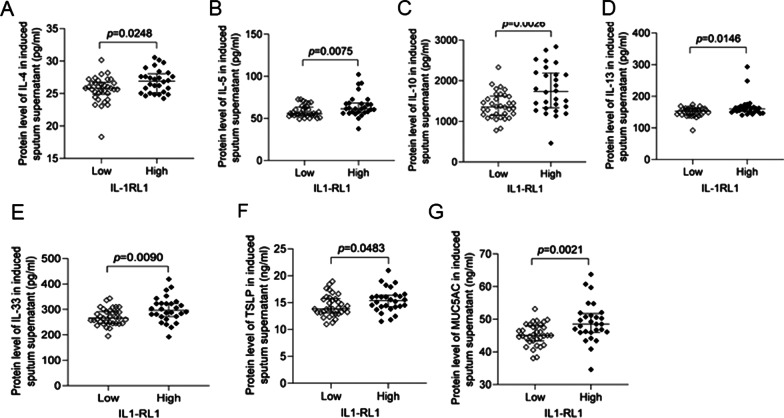
Type 2 inflammation markers of asthma are higher in patients with IL1-RL1-high group. The difference of **A** IL-4; **B** IL-5; **C** IL-10; **D** IL-13; **E** IL-33; **F** TSLP; **G** MUC5AC between IL1-RL1-high and IL1-RL1-low subgroups in induced sputum supernatant

In order to explore the relationship between the expression of IL1-RL1 in sputum supernatant of patients with asthma and type 2 inflammation, we analyzed the correlation between IL1-RL1 and Th2 inflammatory factors, such as IL-4, IL-5, IL-10 and IL-13. In addition, we also detected the expression of IL-33 and TSLP. Because it has been reported that epithelial cells can also release these two factors to affect Th2 reaction, they were considered important initiators of type 2 immunity. And in genome-wide association studies, single nucleotide polymorphisms in the TSLP, IL-33, and IL1-RL1 genes have been identified to be associated with increased atopic and bronchial asthma susceptibility in general [14, 15]. Through analysis, we found that there was a positive correlation between the expression of IL1-RL1 and IL-4 (*r*_*s*_ = 0.3140, *p* = 0.0036), IL-5 (*r*_*s*_ = 0.5221, *p* < 0.0001), IL-10 (*r*_*s*_ = 0.4877, *p* < 0.0001), IL-13 (*r*_*s*_ = 0.4412, *p* < 0.0001), IL-33 (*r*_*s*_ = 0.5125, *p* < 0.0001), TSLP (*r*_*s*_ = 0.4733, *p* < 0.0001) (Fig. 2A–F). Type 2 asthma is characterized by high secretion of mucus, and MUC5AC is the main gel-forming mucin in human airway mucus. We found a significant positive correlation between MUC5AC and IL1-RL1(*r*_*s*_ = 0.5229, *p* < 0.0001) (Fig. 2G). According to the upper quartile of IL1-RL1 expression in healthy controls, patients with asthma were further divided into high IL1-RL1 expression group and low IL1-RL1 expression group. From Fig. 3 we can see that the group with high expression of IL1-RL1 had higher level of IL-4 (*p* = 0.0248), IL-5 (*p* = 0.0075), IL-10 (*p* = 0.0026), IL-13 (*p* = 0.0146), IL-33 (*p* = 0.0090), TSLP (*p* = 0.0483) and MUC5AC (*p* = 0.0021).

### The relationship between IL1-RL1 and Th1 cytokines

**Fig. 4:**
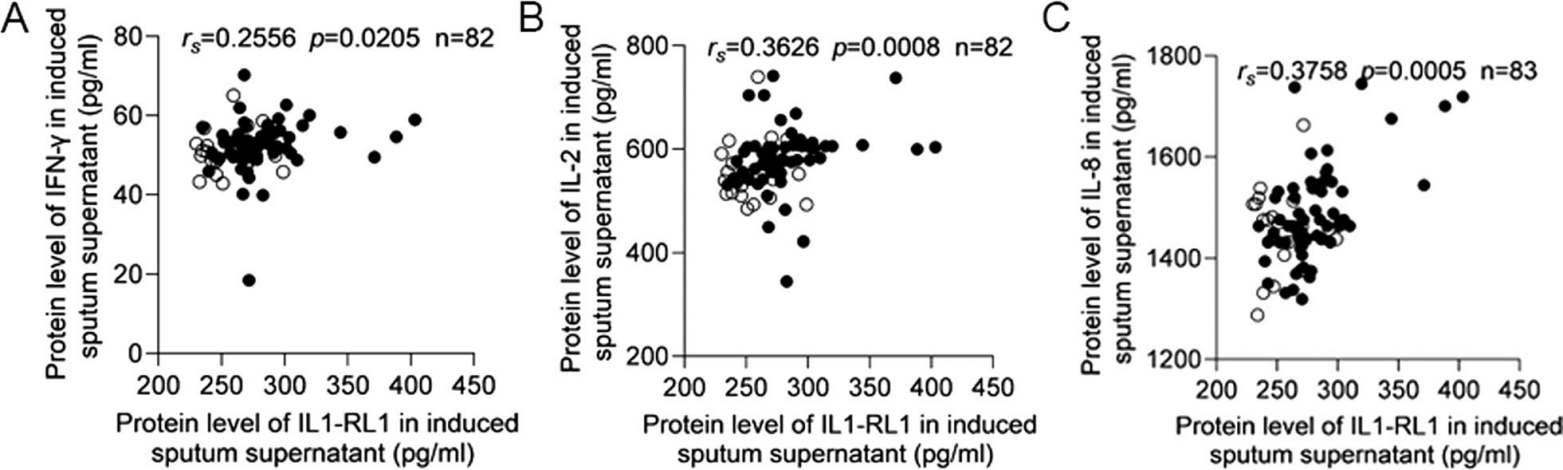
The relationship between IL1-RL1 and Th1 cytokines. The relationship between the protein level of IL1-RL1 in induced sputum supernatant and **A** IFN-γ; **B** IL-2; **C** IL-8

**Fig. 5:**
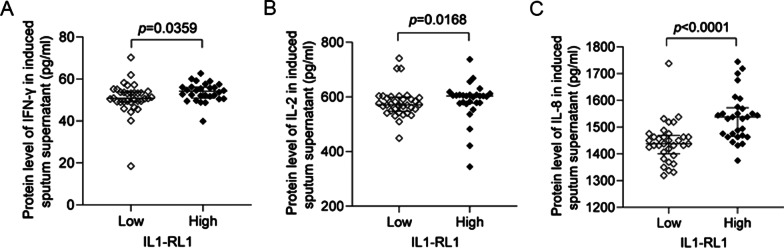
Th1 cytokines of asthma are present in patients with IL1-RL1-high and IL1-RL1-low group. The difference of **A** IFN-γ; **B** IL-2; **C** IL-8 between IL1-RL1-high and IL1-RL1-low subgroups in induced sputum supernatant

Through analysis, we found that IL1-RL1 was also related to the expression of some Th1 cytokines. The result showed that there was a positive correlation between the protein level of IL1-RL1 and IFN-γ, IL-2, IL-8 in the supernatant of sputum (IFN-γ: *r*_*s*_ = 0.2556, *p* = 0.0205, IL-2: *r*_*s*_ = 0.3626, *p* = 0.0008, IL-8: *r*_*s*_ = 0.3758, *p* = 0.0005) (Fig. 4A–C). And asthma patients with high expression of IL1-RL1 had higher level of IFN-γ (*p* = 0.0359), IL-2 (*p* = 0.0168) and IL-8 (*p* < 0.0001) (Fig. 5A–C).

### Expression of IL1-RL1 in serum of patients with asthma

**Fig. 6:**
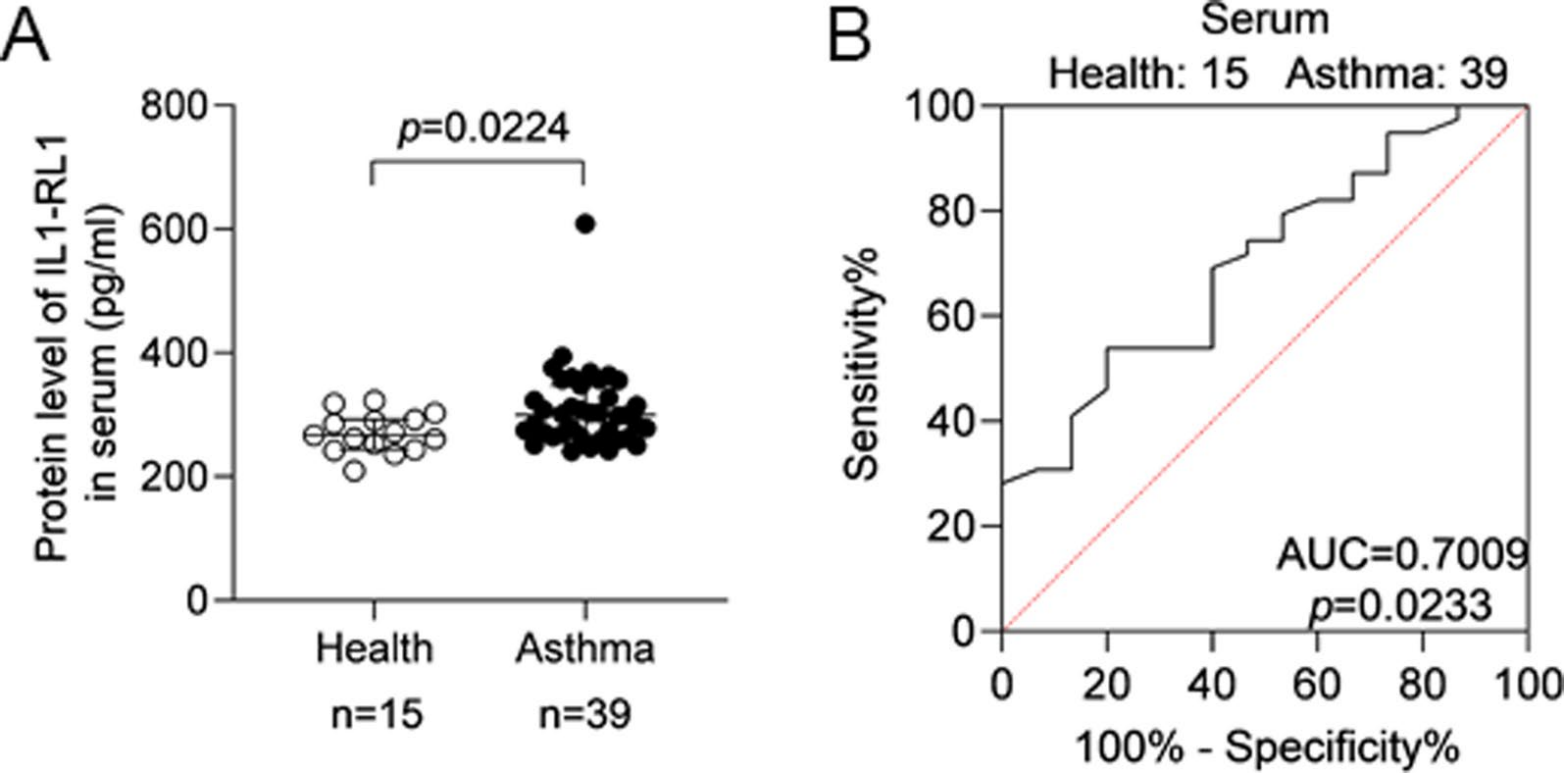
The protein level of IL1-RL1 in serum is increased in asthma. **A** The protein level of IL1-RL1 in serum. **B** The ROC curve of IL1-RL1 in serum

In order to verify the expression of IL1-RL1 in asthma patients from multiple angles, we also detected the expression of IL1-RL1 in serum. ELISA results showed that the protein level of IL1-RL1 in asthma patients’ serum was higher than that in healthy subjects (*p* = 0.0224) (Fig. 6A). The AUC was 0.7009 (*p* = 0.0233) (Fig. 6B). The expression of IL1-RL1 in serum was positively correlated with IgE (*r*_*s*_ = 0.3731, *p* = 0.0193), and there was no correlation with FeNO and EOS# (Additional file 1: Fig. S1A–C). The qRT-PCR results of IL1-RL1 in induced sputum cells were consistent with the expression of sputum supernatant and serum. The mRNA level of IL1-RL1 was higher in asthma patients’ induced sputum cells (*p* = 0.0012), and the AUC was 0.7833 (*p* = 0.0016) (Additional file 1: Fig. S2A, B). The expression of IL1-RL1 in the induced sputum cells was also positively correlated with FeNO (*r*_*s*_ = 0.5919, *p* < 0.0001), IgE (*r*_*s*_ = 0.4144, *p* = 0.0108) and EOS# (*r*_*s*_ = 0.4211, *p* = 0.0044) (Additional file 1: Fig. S2C–E).

### The responsiveness of IL1-RL1 to ICS treatment

**Fig. 7:**
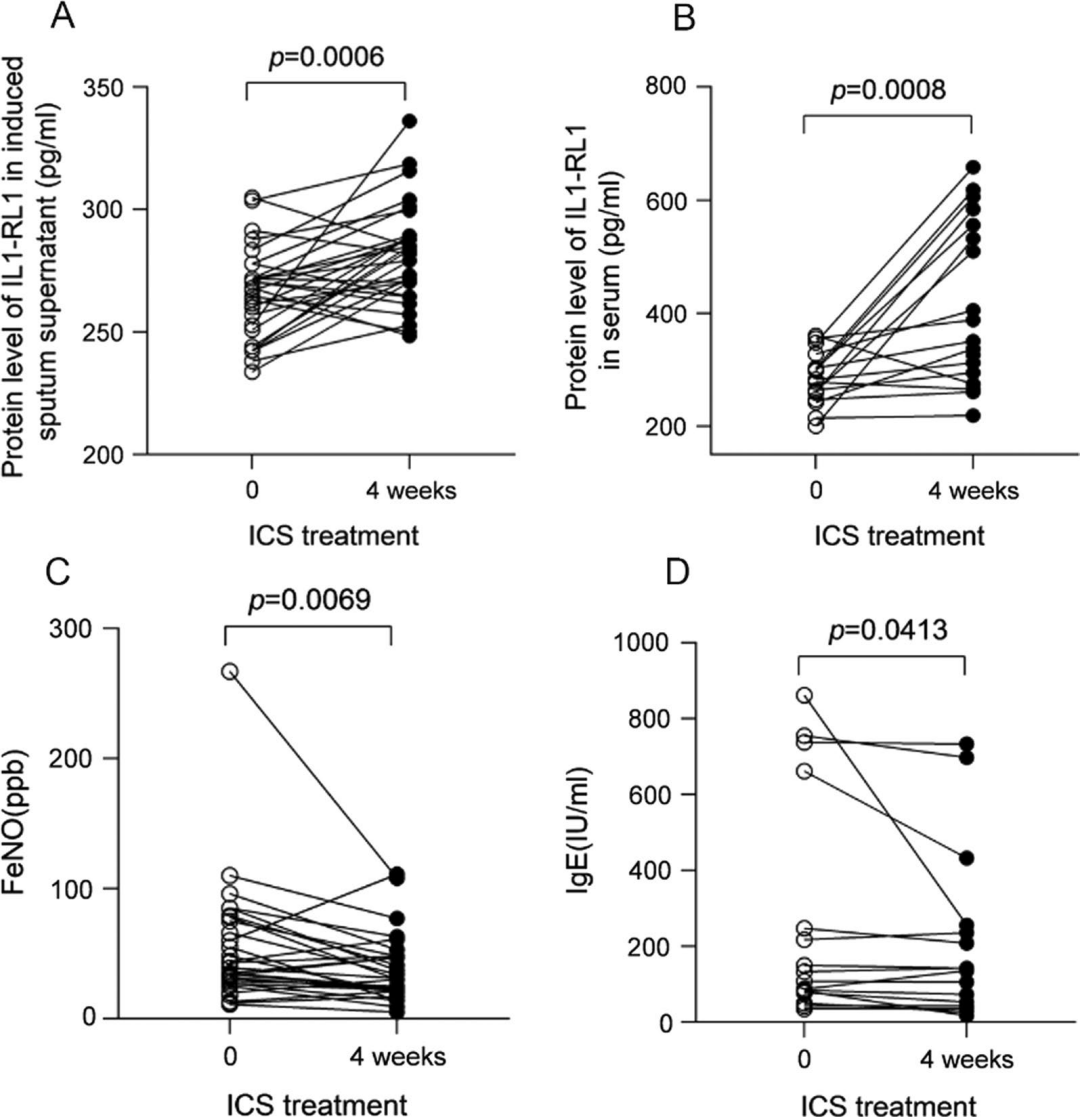
Indicators of patients before and after treatment in asthma patients. Protein levels of IL1-RL1 in **A** induced sputum supernatant and **B** serum; **C** FeNO; **D** IgE before and after ICS treatment for 4 weeks

After 4 weeks of ICS treatment, we found that the expression of IL1-RL1 in induced sputum supernatant and serum of patients with asthma was significantly increased (Fig. 7A–B). FeNO and serum total IgE of patients were also decreased significantly after treatment (Fig. 7C–D). We suspect that IL1-RL1 has a protective effect in patients with asthma.

### The expression of IL1-RL1 in BEAS-2B cell

After IL-4 (10 ng/ml) or IL-13 (20 ng/ml) stimulated BEAS-2B cells for 24 h, we found that the expression of IL1-RL1 was increased through qRT-PCR (Additional file 1: Fig. S3).

## Discussion

In the present study, we found that the expression levels of IL1-RL1 in induced sputum supernatant, serum and induced sputum cells of patients with asthma were higher than healthy controls. IL1-RL1 in the supernatant of induced sputum was positively correlated with FeNO, IgE, EOS#, Th2 and Th1 cytokines. Patients with high IL1-RL1 expression had higher levels of Th2 and Th1 cytokines. The AUC of ROC curve of IL1-RL1 in induced sputum supernatant, induced sputum cells and serum and were 0.6840 (*p* = 0.0034), 0.7833 (*p* = 0.0016), and 0.7009 (*p* = 0.0233), respectively. Four weeks after ICS treatment, the expression of IL1-RL1 in induced sputum supernatant and serum was increased. In vitro, the expression of IL1-RL1 was increased after stimulated by IL-4 or IL-13 for 24 h in BEAS-2B cells.

In 2008, Reijmerink et al. were the first to identify IL1-RL1 as a susceptibility gene for asthma [16]. This observation was replicated in several other candidate-gene and GWAS studies recently summarized by El-Husseini et al. [17]. The IL1-RL1 locus contains a variety of genetic signals, which are independently associated with asthma and allergic susceptibility [3, 18]. Compared with the healthy control group, the expression level of IL-33 in airway epithelial cells of asthma patients was higher. Furthermore, the expression levels were significantly higher in patients with severe asthma than in patients with mild asthma [19–21]. Similarly, compared with healthy controls, high levels of IL1-RL1 and IL-33 were found in serum and sputum of adults and children with acute asthma [22, 23]. Increased IL1-RL1 was also detected in OVA-induced mouse model [20, 24, 25]. We also found that the expression of IL1-RL1 was significantly increased in induced sputum of asthma patients. These results hint that IL1-RL1 may be involved in the development of asthma. Signal mediated by IL33-IL1-RL1 axis participates in the development of allergic diseases by activating ILC2 [26]. Recently, a large number of patient samples and mouse models have shown that ILC2s produce large amounts of Th2 cytokines in response to IL-33/IL1-RL1. In mouse model, transgenic overexpression [27] or administration of IL-33 [28] could increase the number of eosinophils in the airway, increase the expression of Th2 cytokines, increase serum IgE, airway hyper-responsiveness (AHR), and the mucus secretion. On the contrary, IL1-RL1 neutralization decreased airway inflammation, IgE level, Th2 cytokine expression, goblet cell proliferation and AHR [29–31]. Numerous studies have shown that IL-33-IL1-RL1 was involved in Th2 inflammation. In our current study, IL1-RL1 in the supernatant of induced sputum was positively correlated with Th2 cytokines, such as IL-4, IL-5, IL-10, IL-13, IL33 and TSLP. In addition, the level of IL1-RL1 was positively correlated with other eosinophilic indexes such as IgE, FeNO, and EOS#, which suggests that it is involved in type 2 inflammation in asthma.

Beyond that, studies in the past few years have shown that the role of IL-33 is not limited to activating immune cells involved in type 2 immune response. Some studies have shown that IL-33 also plays an important role in the activation of immune cells in type 1 immunity [32]. IL1-RL1 participates in a positive feedback loop driven by IL-33, which acts in Th1 cells. It can promote the production of type 1 cytokines [33]. A dominant Th1 response was also observed in IL1-RL1-deficient mice [34]. And in our research, we found that the expression of IL1-RL1 was positively correlated with some Th1 cytokines (IFN-γ, IL-2 and IL-8). It showed that IL1-RL1 was not only involved in Th2 type inflammation, but also related to Th1 type immune response. To some extent, this is consistent with previous research.

IL-33 is considered to be a driving factor of early inflammation, so some scholars used IL1-RL1 (sST2) to treat OVA-induced airway inflammation in mice, and found that IL1-RL1 can reduce airway inflammation [30]. IL1-RL1 significantly inhibited the increase of IL-4, IL-5, IL-13, IL-25, IL-33, TSLP and IgE in bronchoalveolar lavage fluid of mice induced by OVA, and improved the production of mucus, but had no effect on respiratory tract inflammation driven by Th1. This inhibitory effect on inflammation is caused by IL-33/IL1-RL1 interactions [35]. IL1-RL1 may act as an inhibitor of inflammatory induction of IL-33, thus blocking the pro-inflammatory effect of IL-33 [36–38]. Our data showed that the expression of IL1-RL1 was increased after four weeks of ICS treatment. At the same time, FeNO and IgE of the patients decreased significantly after treatment. An article has shown that cortisol hormone treatment is effective in reducing Th2-type cytokine expression [39]. After cortisol hormone therapy, the levels of IL-4, IL-5, and IL-8 in the sputum of asthma patients were reduced [40, 41], IL-13 expression was decreased in the bronchial epithelium in asthma patients [42]. And the expression level of IL-33 in the serum of asthma patients was also reduced [43]. In addition, the study showed that the expression of MUC5AC was also reduced after treatment with dexamethasone in asthma patients [44], and dexamethasone can also inhibit the expression of IL-2 and IFN-γ [45]. In summary, after cortisol hormonal treatment, our assays showed an increase in IL-1RL1 expression, while the articles points to a decrease in the expression of inflammatory factors. Therefore, we speculate that the elevated IL1-RL1 in asthmatic patients may play a protective and anti-inflammatory role.

Our study also has some limitations. First of all, the population we included was the Chinese population, which lacks diversity. Secondly, we did not detect the cytokines in induced sputum supernatant and serum of patients after 4 weeks ICS treatment. If we can compare the effects of IL1-RL1 on the changes of cytokines before and after treatment, the data will be more comprehensive. Thirdly, we only carried out the analysis on the expression level, and the mechanism of IL1-RL1 in asthma needs to be further studied.

## Conclusion

In summary, we confirmed the expression of IL1-RL1 in asthma patients’ induced sputum supernatant, induced sputum cells and serum and found the correlation between its expression and type 1 and type 2 immune cytokines in patients with asthma, which provided new evidence for the involvement of IL1-RL1 in asthma. IL1-RL1 may be used as a potential diagnostic markers and therapeutic targets in asthma.

## Supplementary Information

**Additional file 1**. The inclusion and exclusion criteria of the subjects and the expression of IL1-RL1 in serum and induced sputum cells of the subjects and in BEAS-2B in vitro.

## Data Availability

The datasets generated and/or analyzed during the current study are not publicly available due to ethical regulation constraints but are available from the corresponding author on reasonable request.

## Abbreviations

EOS#: Peripheral blood eosinophil count
ICS: Inhaled glucocorticoids
FeNO: Fraction of exhaled nitric oxide
GINA: Global Initiative for Asthma
AHR: Airway hyper-responsiveness
