# Supplementary material for: Increased expression of IL1-RL1 is associated with type 2 and type 1 immune pathways in asthma
Source: BMC Immunol. 2022 May 16;23:23. doi: 10.1186/s12865-022-00499-z (PMC9112580; doi:10.1186/s12865-022-00499-z)
Supplement: Supplementary file 1 — Additional file 1. The inclusion and exclusion criteria of the subjects and the expression of IL1-RL1 in serum and induced sputum cells of the subjects and in BEAS-2B in vitro. [file 12865_2022_499_MOESM1_ESM.docx]

**Supplementary figures**


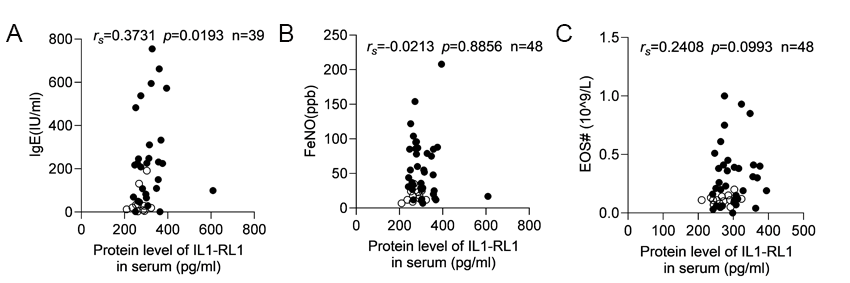


**Fig. S1. The relationship between clinical Indicators and IL-1RL1 in serum.** The relationship between the protein level of IL-1RL1 in serum and (A) IgE; (B) FeNO; (C) EOS#.

**
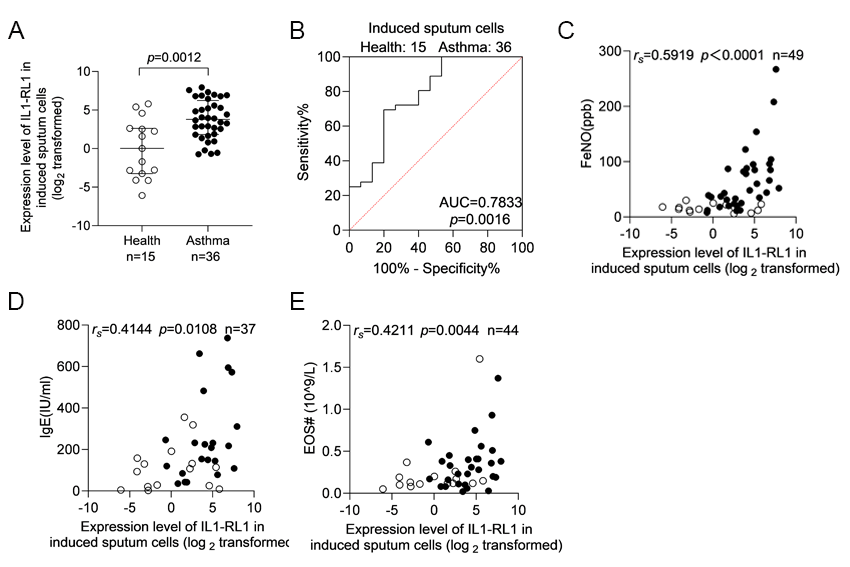
**

**Fig. S2. Induced sputum cells IL-1RL1 is increased and correlates with FeNO, IgE and EOS# in asthma.** (A) The expression level of IL-1RL1 in induced sputum cells. (B) The ROC curve of IL-1RL1 in induced sputum cells. The relationship between the expression level of IL-1RL1 in induced sputum cells and (C) FeNO; (D) IgE; (E) EOS#.


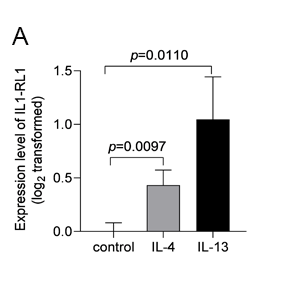


**Fig. S3. The expression level of IL-1RL1 in airway epithelial cells in vitro.** (A) The expression level of IL-1RL1 in BEAS-2B after stimulated with IL-4 and IL-13.

**Table S1**. **Inclusion and exclusion criteria**

|  | Healthy control group | Asthma group |
| --- | --- | --- |
| Inclusion | (1) No obvious abnormality was found in chest X-ray or CT, the results of pulmonary function test were normal;  (2) No history of respiratory diseases such as asthma, chronic obstructive pulmonary disease, pulmonary infection, lung cancer, allergic rhinitis, eczema and other allergic diseases, and no history of autoimmune diseases.  (3) Age ≥ 16 years old. | (1) In line with the diagnostic criteria of Global Initiative for Asthma: ①Variable asthma symptom; ②Variable airflow limitation test; ③Patients with variable asthma symptoms and those who meet any of the variable airflow limitation tests can be diagnosed with asthma. Atypical asthma without obvious wheezing symptoms should have at least one of the above tests. (2) Diagnosed with asthma for the first time.   1. Age ≥ 16 years old. |
| Exclusion | - | (1) Complicated with other respiratory diseases or immune system related diseases;  (2) Associated with circulatory, digestive, urinary and neurological systems diseases;  (3) Pregnant or lactating women;  (4) Long-term or nearly 1 month has received standard drugs or anti-infective treatment. |

Detailed inclusion and exclusion criteria for healthy controls and patients with asthma.
